# Integrity at stake: confronting “publish or perish” in the developing world and emerging economies

**DOI:** 10.3389/fmed.2024.1405424

**Published:** 2024-07-17

**Authors:** Jorge Vasconez-Gonzalez, Juan S. Izquierdo-Condoy, Patricio Naranjo-Lara, Miguel Ángel Garcia-Bereguiain, Esteban Ortiz-Prado

**Affiliations:** One Health Research Group, Universidad de Las Américas, Quito, Ecuador

**Keywords:** authorship for sale, ethical issues, publishing, research integrity, science production, low income

## Abstract

The scientific community faces significant ethical challenges due to the “publish or perish” culture, particularly in developing and emerging economies. This paper explores the widespread unethical practices in scientific publishing, including the sale of authorships, the proliferation of “paper mills,” and the misuse of artificial intelligence to produce fraudulent research. These practices undermine the integrity of scientific research, skew publication metrics, and distort academic rankings. This study examines various instances of academic fraud, emphasizing the impact on low-income countries, with specific cases from Latin America. Recommendations include stricter verification of authorship, disciplinary measures for scientific fraud, and policies promoting transparency and accountability in research. Addressing these challenges is crucial for maintaining the integrity and credibility of scientific endeavors globally.

From its inception, the scientific method has been instrumental in the advancement of various societies, serving not only to resolve complex problems but also as a fundamental tool for the generation of knowledge through theories and propositions (1–3). These scholarly contributions have significantly impacted the realms of invention and have markedly improved the quality of life across the globe. Over the last centuries, scientific publishing has witnessed a profound evolution, expanding from a modest collection of 10 journals in the 17th century to an extensive network of over 100,000 journals by the close of the 20th century (4). This remarkable growth highlights the essential role that publishing plays in the progress of science; facilitating the widespread dissemination of groundbreaking innovative ideas, findings, and theories (5). Consequently, the act of publishing has become a pivotal component of the activities that researchers must fulfill, with universities increasingly regarding scientific papers as critical metrics for evaluation and ranking within the academic community (6, 7).

In this landscape, however, the pressures of “publish or perish” have catalyzed the emergence of unethical practices within the academic community (8). It is well known by the scientific community worldwide that there are several forums on the internet where the trade of authorships happens. Furthermore, the so-called paper mills, are organizations that use artificial intelligence (AI) and other tools to produce large amounts of publications with the sole purpose of offering authorship for sale for as little as $200 USD without making any substantive contribution to the scientific article or publication in question (9). Some websites have been identified that negotiate authorships based in Russia, Iran, or Latvia; some have even mentioned having published more than 12,000 articles and offering to be the main author of an article for 2,000 euros (10).

In this context, social networks have become a powerful tool for the sale of articles and theses (7–9). Consequently, this undermines the quality and integrity of the research community, jeopardizes the credibility of genuine academic work, and corrupts the whole academic system, where excellence is evaluated by publication records (6). These platforms have become centers of unethical commodification of academic credentials (9), which aggravates the problems of integrity in scientific publication and manages to alter the metrics and rankings of scientific production.

Recent revelations, such as the case of a Spanish chemist who astonishingly published one article every 37 h; or the case of a Japanese psychiatrist who in 1 year published 115 articles, have brought to light instances of academic fraud that have shaken the scientific community (11, 12). Another striking case was that 78 journals were notified of approximately 300 articles by two Japanese doctors that showed signs of manufacturing and other ethical failures; almost half of those articles were retracted (13). The emergence of “paper mills” offering scientific articles, data, and ghostwriting services for a fee has exposed long-standing, but only recently acknowledged, practices that compromise the integrity of scientific publishing (14–16).

According to a June 2022 report from the Publications Ethics Committee, it is estimated that 2% of articles submitted to journals come from paper mills, with some analyses suggesting that this figure could be as high as 20% (17). An analysis of 2.85 million studies published in 2022 found that approximately 2.2% appeared to be paper mill studies (17). Regarding the use of AI, more than 100 articles have been identified as likely partially written by ChatGPT (18). Since the launch of this tool, there has been a 72% increase in articles potentially written by AI, despite evidence that AI can commit data falsification and fabricate non-existent results (18–20). The consequences of such practices in biomedical research include scientific fraud, misleading statistics, content saturation, fraud in the application of funds, and increased pressure on legitimate researchers (21).

The undeniable importance of publishing scientific articles for the advancement of knowledge brings with it both financial and non-financial benefits, including the promotion of new scientific collaborations, career advancement opportunities, the securing of permanent positions, and the facilitation of financial agreements (22). These benefits not only serve the individual researchers but also enhance the scientific reputation of their affiliated institutions, attracting potential collaborators, generating funds, and accruing prestige (23).

In response to the evolving landscape of scientific publishing, universities worldwide have intensified their efforts to climb the academic rankings. Rankings such as Scimago, Shanghai Ranking, and Times Higher Education, are considered a measure of quality worldwide (24). Those rankings include publication records in the variables to score any institution, and they also have great visibility in the field of higher education policies, the strategies of their institutional development, and their impact on all media (24). This ambition is driven by the potential to unlock greater opportunities for accessing both international and national competitive funding, thereby incentivizing the recruitment of scientists capable of drafting grant proposals that can translate into substantial financial returns and underpin the foundation for scientific development (22). Notable examples include powerhouse nations such as the United States and China, which allocate significant resources toward basic research endeavors.

This backdrop has compelled many scientists to navigate the precarious “publish or perish” landscape, a dynamic that not only induces stress among researchers but also fosters the emergence of unethical practices detailed above (25). Added to this is the multiple affiliations that came to light with the suspension of one of the researchers at the University of Córdoba in Spain for affiliating himself with institutions such as King Saud University and the People’s Friendship University of Russia, which underscores the broader issue of researchers falsely claiming affiliations to artificially boost the global rankings of these institutions (11). After this, several cases have been known in which universities in Saudi Arabia changed their main place of work in the database, such as a Catalan chemist who was offered 70,000 euros per year or a Spanish physicist who accepted a National Research Award for Young People scholarship (26).

This situation has reached all regions, including South America. In Peru, reports have surfaced of university faculty members from both public and private institutions paying up to USD 500 to be included as authors in scientific research without having made any substantive contributions (9).

In the case of Ecuador, there are universities where some of the journals it publishes are included in the list of discontinued journals in Scopus for “Publication Concerns” (27). This phenomenon, facilitated by social media platforms and groups such as “PublishScorpus,” underscores a systemic problem within the academic research field, where the pressure to publish can lead to ethical compromises and threaten academic integrity. Such practices, while enhancing individual resumes, undermine the credibility of the scientific enterprise and highlight the urgent need for reform.

These unethical practices do not merely undermine the integrity of the research environment; they also result in substantial tangible consequences. These impacts are far-reaching and affect various aspects of the scientific community. They waste valuable resources, jeopardize funding opportunities, undermine scientific integrity, and have long-term detrimental effects on the reputation and productivity of research institutions. A comprehensive summary of these tangible effects is presented in Figure 1, illustrating the severity and breadth of the issues caused by fraudulent research practices (28–30).

**Figure 1:**
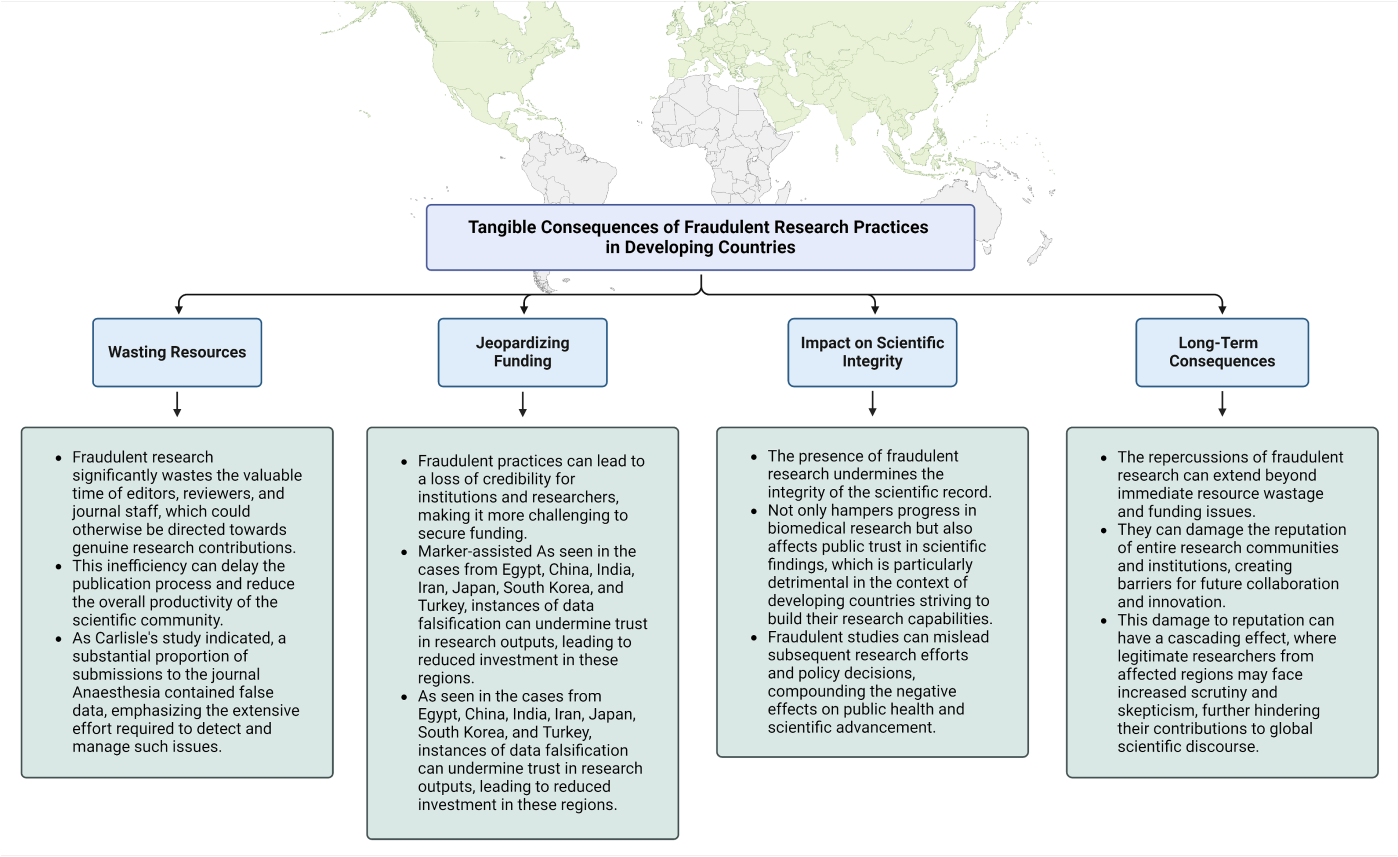
Tangible consequences of fraudulent research practices in developing countries (28–30).

The responsibility to address these challenges extends beyond those directly involved to encompass the entire scientific community. The widespread targeting of scientists by unethical publishers and predatory journals, particularly in regions such as South America and parts of the Middle East, necessitates a collective effort to promote transparency, accountability, and ethical conduct in scientific publishing (31, 32). By confronting these unethical practices and fostering a culture of integrity, we can protect the foundational principles of scientific research and ensure its continued role in driving human progress into the 21st century and beyond.

Therefore, it is incumbent upon all stakeholders within the scientific community, especially universities, hospitals, and research institutes, to diligently scrutinize publications and affiliations that could artificially enhance profiles. Scientific journals, in turn, must rigorously ensure that authorship is rightfully attributed based on genuine contributions, such as verifying that the academic training of the authors is related to the topic or area of research. For example, in Peru, doctors in education appeared in articles on nanomaterials or medical research (33, 34).

Another key element in maintaining the integrity of scientific publications is the role of universities, which must institute disciplinary measures for scientific fraud (35). Governing bodies in charge of controlling research activities should establish committees responsible for retracting scientific journals with repeat fraudulent publications and establish channels for reporting unethical scientific conduct. Additionally, governments must generate policies and establish disciplinary measures (35).

By doing so, we can safeguard scientific integrity, uphold fundamental ethical principles, and mitigate the potential for misinformation that can mislead the broader scientific community and the public at large. Addressing these pressing issues not only protects the sanctity of scientific research but also affirms its indispensable contribution to society.

## Data availability statement

The original contributions presented in the study are included in the article/supplementary material, further inquiries can be directed to the corresponding author.

## Author contributions

JV-G: Investigation, Methodology, Project administration, Writing – review & editing. JI-C: Data curation, Investigation, Methodology, Project administration, Validation, Writing – original draft, Writing – review & editing. PN-L: Investigation, Methodology, Validation, Writing – review & editing. MG-B: Validation, Writing – review & editing. EO-P: Conceptualization, Investigation, Supervision, Validation, Writing – original draft, Writing – review & editing.
